# Development and validation of predictive model for long-term hospitalization, readmission, and in-hospital death of patients over 60 years old

**DOI:** 10.31744/einstein_journal/2022AO8012

**Published:** 2022-06-14

**Authors:** Maria Luiza Monteiro Costa, Ana Carolina Cintra Nunes Mafra, Maysa Seabra Cendoroglo, Patrícia Silveira Rodrigues, Milene Silva Ferreira, Stephanie A. Studenski, Fábio Gazelato de Mello Franco

**Affiliations:** 1 Hospital Israelita Albert Einstein São Paulo SP Brazil Hospital Israelita Albert Einstein, São Paulo, SP, Brazil.; 2 Universidade Federal de São Paulo São Paulo SP Brazil Universidade Federal de São Paulo, São Paulo, SP, Brazil.; 3 University of Pittsburgh Pittsburgh PA United States University of Pittsburgh, Pittsburgh, PA, United States.; 4 Faculdade Israelita de Ciências da Saúde Albert Einstein Hospital Israelita Albert Einstein São Paulo SP Brazil Faculdade Israelita de Ciências da Saúde Albert Einstein, Hospital Israelita Albert Einstein, São Paulo, SP, Brazil.

**Keywords:** Aging, lenght of stay, Long-term care, Patient readmission, Hospital mortality, Hospitalization, Logistic models

## Abstract

**Objective:**

To develop and validate a high-risk predictive model that identifies, at least, one common adverse event in older population: early readmission (up to 30 days after discharge), long hospital stays (10 days or more) or in-hospital deaths.

**Methods:**

This was a retrospective cohort study including patients aged 60 years or older (n=340) admitted at a 630-beds tertiary hospital, located in the city of São Paulo, Brazil. A predictive model of high-risk indication was developed by analyzing logistical regression models. This model prognostic capacity was assessed by measuring accuracy, sensitivity, specificity, and positive and negative predictive values. Areas under the receiver operating characteristic curve with 95% confidence intervals were also obtained to assess the discriminatory power of the model. Internal validation of the prognostic model was performed in a separate sample (n=168).

**Results:**

Statistically significant predictors were identified, such as current Barthel Index, number of medications in use, presence of *diabetes mellitus*, difficulty chewing or swallowing, extensive surgery, and dementia. The study observed discrimination model acceptance in the construction sample 0.77 (95% confidence interval: 0.71-0.83) and good calibration. The characteristics of the validation samples were similar, and the receiver operating characteristic curve area was 0.687 (95% confidence interval: 0.598-0.776). We could assess an older patient’s adverse health events during hospitalization after admission.

**Conclusion:**

A predictive model with acceptable discrimination was obtained, with satisfactory results for early readmission (30 days), long hospital stays (10 days), or in-hospital death.

## INTRODUCTION

Population aging impacts society in many ways including health, economics, politics, and social aspects.^(1,2)^ In Brazil, demographic transition has happened faster than in developed countries. The Brazilian Institute of Geography and Statistics (IBGE - *Instituto Brasileiro de Geografia e Estatística*) estimates that the number of older adults will exceed the number of children and adolescents in 2040.^(3)^

Substantial improvements in the medical and technological fields have contributed to increasing life expectancy, changing the epidemiological profile.^(4,5)^ The increased frequency of chronic diseases leads to longer hospital stays, increased risk of readmissions, institutionalization, and physical dysfunction after hospital discharge, which also increase mortality.^(6)^ Thus, the big challenge is to identify vulnerable patients to propose interventions that can reduce undesirable outcomes. Evidence from other countries suggests that hospital screening for geriatric issues helped with discharge planning, decreased mortality and readmissions.^(7)^ Functional status is one of the most important condition that must be preserved,^(8)^ but it is common sense that the multiple domains of geriatric assessment^(9)^ should be considered. Some of the promising tools are the Multidimensional Prognostic Index (MPI)^(10)^ and the Charlson Comorbidity Index (CCI)^(11)^ that are associated with mortality, institutionalization, and readmission. In Brazil, such risk screening is not yet part of the assessment of public or private hospitals.^(12,13)^

## OBJECTIVE

To construct a predictive model that identifies at least one of the following events: death during hospitalization, early readmission up to 30 days after discharge, or long hospital stay (10 days or more); and to validate the model in an older adults’ population.

## METHODS

This was a retrospective cohort study involving 508 patients admitted at a private tertiary hospital (630 beds) in São Paulo city, Brazil. The inclusion criteria were patients aged ≥60 years, hospitalized in clinical and surgery wards. The exclusion criteria were patients admitted in psychiatric and intensive care wards. Patients were followed until discharge.

We used a data base of a previous cohort study, collected from March 2014 to June 2015, and published in 2019.^(14)^ The time interval between admission and data collection of up to 72 hours was defined to avoid loss of information in cases of patients with rapid therapeutic response and discharge or death before 7 days. A single trained professional assessed the patients using the Barthel Index score^(15,16)^ as a measure of functional capacity 30 days before admission and at the time of admission. The Barthel Index belongs to the area of assessment of activities of daily living (ADLs) and measures functional independence in personal care, mobility, ambulation, and continence. Ten tasks are evaluated: eating, bathing, dressing, personal hygiene, bowel, and bladder control, using the toilet, chair-to-bed transferring, walking, and stairs. The instrument scores each item according to the patient’s performance ability to perform tasks independently, with moderate assistance or total dependence. A score is assigned to each category, depending on the time and assistance required for each patient. The classification ranges from 0 to 100, at intervals of five points, with higher scores indicating more independence.^(16)^

A cognitive assessment was performed by the Short Portable Status Questionnaire (SPMSQ), also at admission.^(17,18)^ It consists of a 10-item questionnaire that measures the presence of cognitive impairment, considering general knowledge and personal information. Patients are asked questions such as the date, their telephone number, address, age, birthplace, maiden name, the current president’s name, and to do subtractions (subtracting 3 from 20 sequentially, up to six times). Four categories are established: normal cognitive functioning, moderate impairment, severe impairment, and unable to respond.^(17)^

Clinical and demographic information was also included. Concomitantly, information concerning the remaining variables was extracted from medical records.

The two samples (keeping a 2:1 ratio), construction and validation, were described separately and compared by Fisher’s exact χ^2^ and Mann-Whitney tests. Within the construction sample (n=340), simple models were initially adjusted and then, using the stepwise method, the multiple model was obtained, to maintain only significant variables to the model (p<0.005). To measure the goodness of fit of the statistical models, the study used a model comparison and variable selection following two directions: including and excluding variables one by one according to the Akaike information criterion (AIC), where lower AIC values represent greater quality and simplicity.^(19)^ The quality of the final model was evaluated by analyzing the standard errors of the estimated coefficients, fit quality graphs, and the Nagelkerke’s R^2^ determination coefficient^(20)^ to measure how much the independent variables included can explain the phenomenon studied (the larger the measure, the more complete and explanatory the model), and variance inflation factor to ensure collinearity between the independent variables considered in the proposed prognostic model.^(21)^ Hosmer and Lemeshow test was conducted, and the Brier score was obtained related to the global predictive capacity performance of the model.^(20)^ The smaller the difference between estimated and observed, the more informative the model is considered. Measures between 0 and 0.25 are considered ideal.^(20)^

This study considered long hospital stays (10 days or more), early readmission (up to 30 days after discharge), and in-hospital death as dependent variables for the model.^(14,22-27)^

The prognostic capacity of this model and its internal validation were assessed by measuring accuracy, sensitivity, specificity, positive and negative predictive values. Areas under the receiver operating characteristic (ROC) curve with 95% confidence intervals (95%CI) were also obtained to assess the model discriminatory power, as well as internal validation of the prognostic model with separate sample (n=168).^(22,27)^

### Ethics approval and consent to participate

The Ethics Committee of the *Hospital Israelita Albert Einstein* (HIAE) approved this study opinion # 3.625.696, CAAE: 61145816.5.0000.0071. All participants signed an informed consent form prior to participation. The patients with no conditions to sign their consent were represented by their legal guardians.

## RESULTS

The study comprises data from 508 patients randomly divided into two samples: 340 for the construction model and 168 for the internal validation. Shapiro-Wilk tests confirmed that the samples are similar, and their distributions are not symmetrical.

Table 1 presents the outcome of interest and clinical, functional, and cognitive characteristics of the construction and validation population.

**Table 1:** Description of interest outcome and clinical/demographic profile

Factors	Construction (n=340)	Validation (n=168)
Hospital outcome		
Discharge	97.9 (333)	98.8 (166)
Death	2.1 (7)	1.2 (2)
Permanence (days)		
Median [1-3 quartiles]	4.00 [3.00-7.00]	3.00 [2.00-6.00]
Permanence over 10 days	12.1 (41)	14.9 (25)
Time until readmission, days (n=143)		
Median [1-3 quartiles]	32.00 [10.00-59.00]	28.50 [6.00-50.25]
Readmission in 30 days	10.9 (37)	13.1 (22)
Sex		
Women	44.4 (151)	44.6 (75)
Men	55.6 (189)	55.4 (93)
Number of diagnostics		
Median [1-3 quartiles]	3.00 [2.00-4.00]	3.00 [2.00-4.00]
Stroke	5.9 (20)	8.3 (14)
Chronic pulmonary obstructive disease	2.6 (9)	4.2 (7)
Neoplasia	22.4 (76)	26.2 (44)
Dialytic chronic renal failure	2.4 (8)	4.2 (7)
Congestive heart failure	7.4 (25)	13.1 (22)
DM	27.4 (93)	30.4 (51)
Dementia	6.8 (23)	6.0 (10)
Hepatopathy	3.8 (13)	3.6 (6)
Acquired immunodeficiency disease syndrome	0.0 (0)	0.0 (0)
Coronary insufficiency	12.1 (41)	8.9 (15)
Total cognitive score		
Median [1-3 quartiles]	0.00 [0.00-1.00]	0.00 [0.00-1.00]
Normal cognitive functioning	77.9 (265)	78.0 (131)
Moderate impairment	5.3 (18)	6.5 (11)
Severe impairment	3.2 (11)	2.4 (4)
Unable to respond	13.5 (46)	13.1 (22)
Last month’s Barthel Index		
Median [1-3 quartiles]	95.00 [80.00-100.00]	97.50 [85.00-100.00]
Independence	45.6 (155)	50.0 (84)
Very mild dependence	17.4 (59)	14.3 (24)
Moderate dependence	19.1 (65)	22.6 (38)
Severe dependency	10.6 (36)	6.0 (10)
Total dependence	7.4 (25)	7.1 (12)
Actual month’s Barthel Index		
Median [1-3 quartiles]	85.00 [60.00-100.00]	85.00 [60.00-100.00]
Independence	26.5 (90)	26.2 (44)
Very mild dependence	7.9 (27)	7.7 (13)
Moderate dependence	37.9 (129)	40.5 (68)
Severe dependency	17.9 (61)	16.7 (28)
Total dependence	9.7 (33)	8.9 (15)
Admission factors or patient status		
Hemoglobin		
Median [1-3 quartiles]	12.70 [11.57-13.80]	12.80 [11.50-13.90]
Medications number		
Median [1-3 quartiles]	5.00 [3.00-8.00]	6.00 [4.00-8.00]
Hospitalization in 6 months		
Median [1-3 quartiles]	0.00 [0.00-1.00]	0.00 [0.00-1.00]
Origin		
Long permanence institution	0.9 (3)	0.0 (0)
Residence	99.1 (337)	100.0 (168)
Live alone	18.8 (64)	16.1 (27)
Delirium	10.0 (34)	10.7 (18)
Incontinence	37.4 (127)	35.7 (60)
Falls	20.9 (71)	20.8 (35)
Nutritional risk	65.6 (223)	67.3 (113)
Weight loss	1.2 (4)	0.0 (0)
Nutritional education	0.9 (3)	1.2 (2)
Difficulty in chewing/Swallowing	3.2 (11)	2.4 (4)
Lowering awareness	2.4 (8)	3.0 (5)
Fasting more than 72 hours	1.8 (6)	1.8 (3)
Diarrhea	1.2 (4)	1.8 (3)
Newly diagnosed/decompensated DM	3.5 (12)	3.0 (5)
Nausea/Vomiting	0.3 (1)	0.6 (1)
Risk or presence of hypoglycemia	21.2 (72)	28.0 (47)
Extensive surgery*	4.1 (14)	0.6 (1)
Polytrauma/Sepsis/Ventilation	0.3 (1)	1.2 (2)
Ulcer	7.4 (25)	11.9 (20)
Food allergy or specific diet	49.1 (167)	47.6 (80)
Enteral/Parenteral nutrition	2.1 (7)	3.6 (6)

* p value <0.05. Categorical measurements are presented by percentage accompanied by absolute frequency in parentheses.

DM: *diabetes mellitus*.

The population of this study consists mostly of older adults with a 4-day median stay that remain under 10 days and have a low mortality rate. Its readmission rate is relevant, up to 32 days after discharge. It is formed by a slightly larger number of male patients than female. They came from their houses and few of them live alone. Most were considered independent and with mild dependence by the Barthel scale. Interestingly, in the median, a large contingent has at least moderate dependence. They presented normal cognitive conditions, and a few have a diagnosis as one of the reasons for hospitalization. However, the incidence of dementia is significant. At least half of the patients had up to three diagnoses, whereas the most prevalent was *diabetes mellitus* (DM). Swallowing/chewing difficulty and polypharmacy are also important associated factors found in this study. According to this profile, if we transpose the risk factors in the aging process timeline, they are strongly limiting and important for the studied outcomes.

### Predictive model

In the univariate models (Table 2) the following factors associated with high risk were noted number of diagnoses, DM, dementia, number of medications, delirium, difficulty chewing or swallowing, lowering of consciousness, ulcer, cognitive score, and the current and last month’s Barthel Index. The functional status at hospital current moment was considered because when compared to the measure of functional capacity 30 days before admission, no statistically significant difference was observed.

**Table 2:** Clinical admission and demographic factors associated with high risk

Factors	High risk		p value
	No (n=271)	Yes (n=79)	
Gender			
Women	42.8 (116)	50.7 (35)	
Men	57.2 (155)	49.3 (34)	0.238
Diagnosis number			
Median [1-3 quartiles]	3.00 [2.00-4.00]	3.00 [2.00-4.00]	0.006
Stroke	4.1 (11)	13.0 (9)	0.007
Chronic pulmonary obstructive disease	2.6 (7)	2.9 (2)	0.884
Neoplasia	22.9 (62)	20.3 (14)	0.645
Dialytic chronic renal failure	3.0 (8)	0.0 (0)	-
Congestive heart failure	5.9 (16)	13.0 (9)	0.048
DM	24.4 (66)	39.1 (27)	0.015
Dementia	4.1 (11)	17.4 (12)	<0.001
Hepatopathy	4.1 (11)	2.9 (2)	0.655
AIDS	0.0 (0)	0.0 (0)	-
Coronary insufficiency	11.8 (32)	13.0 (9)	0.779
Hemoglobin			
Median [1-3 quartiles]	12.70 [11.65-13.80]	12.30 [11.10-13.50]	0.276
Medications number			
Median [1-3 quartiles]	5.00 [3.00-8.00]	7.00 [5.00-9.00]	<0.001
Hospitalization in 6 months			
Median [1-3 quartiles]	0.00 [0.00-1.00]	0.00 [0.00-1.00]	0.187
Origin			
Long permanence institution	0.7 (2)	1.4 (1)	
Residence	99.3 (269)	98.6 (68)	0.58
Live alone	18.1 (49)	21.7 (15)	0.488
Delirium	7.7 (21)	18.8 (13)	0.008
Incontinence	33.2 (90)	53.6 (37)	0.002
Falls	20.3 (55)	23.2 (16)	0.598
Nutritional risk	62.0 (168)	79.7 (55)	0.007
Weight loss	1.5 (4)	0.0 (0)	-
Nutritional education	0.4 (1)	2.9 (2)	0.09
Difficulty in chewing/Swallowing	1.5 (4)	10.1 (7)	0.002
Lowering awareness	0.7 (2)	8.7 (6)	0.002
Fasting + 72 hours	2.2 (6)	0.0 (0)	-
Diarrhea	1.1 (3)	1.4 (1)	0.814
Newly diagnosed/decompensated DM	3.0 (8)	5.8 (4)	0.262
Nausea/Vomiting	0.0 (0)	1.4 (1)	-
Risk or presence of hypoglycemia	19.2 (52)	29.0 (20)	0.078
Extensive surgery*	3.3 (9)	7.2 (5)	0.153
Polytrauma/Sepsis/Ventilation	0.0 (0)	1.4 (1)	-
Ulcer	5.2 (14)	15.9 (11)	0.004
Food allergy or specific diet	46.9 (127)	58.0 (40)	0.101
Enteral/Parenteral nutrition	1.8 (5)	2.9 (2)	0.585
Total cognitive score			
Median [1-3 quartiles]	0.00 [0.00-1.00]	0.00 [0.00-1.00]	0.822
Normal cognitive functioning	83.4 (226)	56.5 (39)	
Moderate impairment	5.2 (14)	5.8 (4)	0.395
Severe impairment	3.0 (8)	4.3 (3)	0.267
Unable to respond	8.5 (23)	33.3 (23)	<0.001
Association between high risk and the current and last month’s Barthel Index			
Current - last month Barthel Index			
Median [1-3 quartiles]	0.00 [-20.00-0.00]	0.00 [-10.00-0.00]	0.476
Worse Barthel Index			
No	50.6 (137)	58.0 (40)	
Yes	49.4 (134)	42.0 (29)	0.272

* p value <0.05. The symbol “-“ indicates that it was not possible to conduct an appropriate adjustment due to the lack of representativeness of any crossing of information. Categorical measurements are presented by percentage accompanied by absolute frequency in parentheses. P values obtained by simple logistic adjustments.

DM: *diabetes mellitus*.

Table 3 shows the results of multiple-variable regression modeling. Statistically significant high-risk predictors of 30-day readmission, lengths of stay (LOS) and in-hospital death indicated as determinants are: the Barthel Index at admission, number of medications in use, presence of DM, and difficulty for chewing or swallowing. Patients who underwent extensive surgery or had dementia diagnoses were not considered as a significantly associated factor in the multiple-variable model, but they were still maintained because of their contribution to the outcome discrimination.

**Table 3:** High-risk multiple predictive logistic model

Variables	Estimated coefficient	Odds ratios (95%CI)	p value
Intercept	-3.2305		<0.001
Current Barthel Index			
Independence (reference)		1.00	
Mild or moderate dependence	0.6608	1.94 (0.81-4.64)	0.138
Severe dependence	1.4876	4.43 (1.70-11.54)	0.002
Total dependence	1.4748	4.37 (1.40-13.60)	0.011
Number of medicines (U)	0.0896	1.09 (1.01-1.18)	0.022
Chewing/Swallowing difficulty (Present)	1.9226	6.84 (1.73-27.03)	0.006
DM (Present)	0.6868	1.99 (1.05-3.77)	0.035
Extensive surgery (Present)	1.2131	3.36 (0.96-11.85)	0.059
Dementia (Present)	0.8903	2.44 (0.91-6.53)	0.077

95%CI: 95% confidence interval. DM: *diabetes mellitus.* n=340.

The risk score was obtained according to the following equation: 1 / (1 + e ^risk score^). The risk score is represented by the logit function obtained by the logistic model using the estimated coefficients presented in table 3.

Risk score=-3.2305 + (0.6608 x current Barthel Index indicating mild or moderate dependence) + (1.4876 x current BI indicating severe dependence) + (1.4748 x current IB indicating total dependence) + (0.0896 x number of medications being used) + (1.9226 x chewing or swallowing difficulty) + (0.6868 x DM) + (1.2131 x extensive surgery) + (0.8903 x dementia), where all indicator variables were coded as 0 for no and 1 for yes, and the number of medications should be replaced by the observed number.

**Figure 1:**
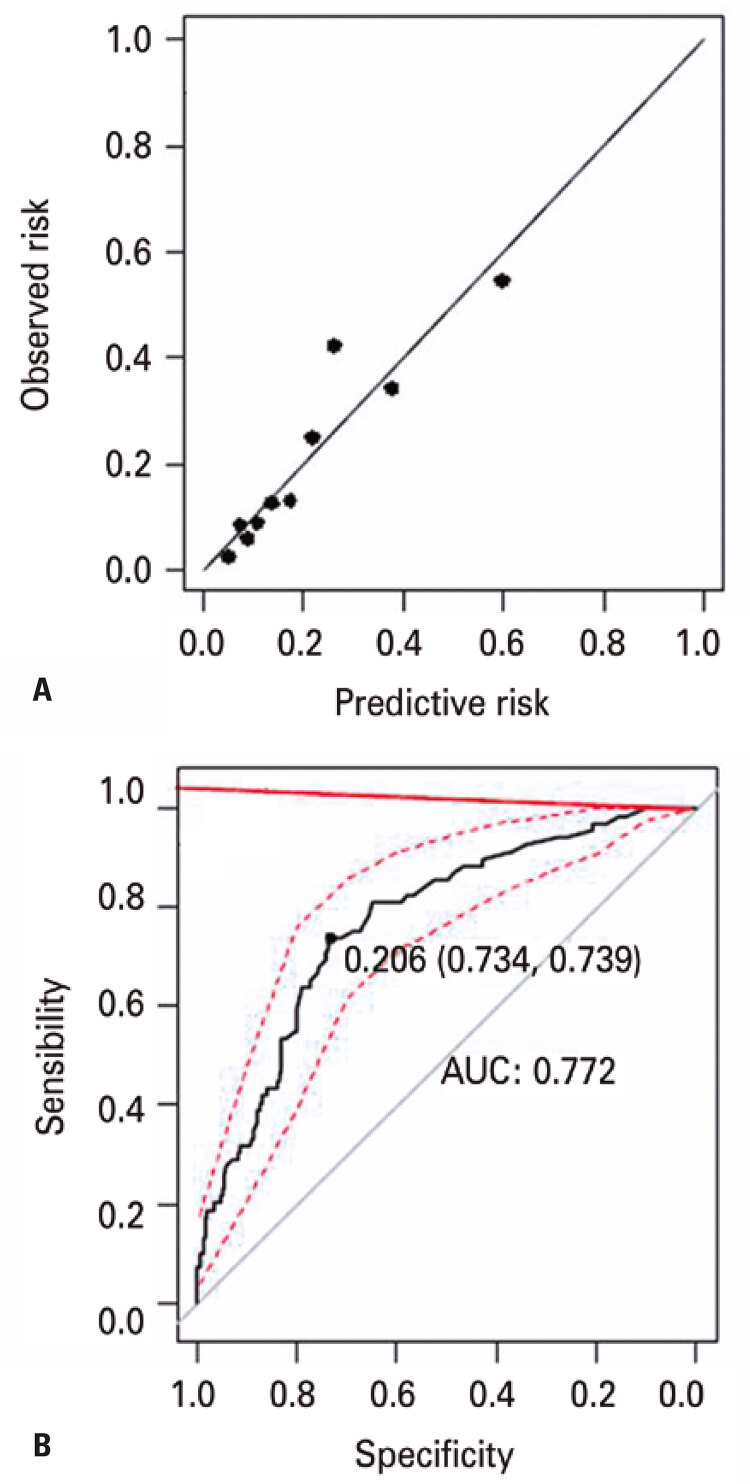
Model fit quality analysis charts. (A) Hosmer and Lemes how calibration graphic; (B) ROC curve

Brier score was 0.14 and indicates that the model can be considered informative and the Hosmer-Lemeshow test indicated goodness of fit quality which show an ideal line and symmetric distribution (Figure 1A).

n=340. AUC: area under the curve.

It was observed an area under the ROC curve of 0.77 (95%CI: 0.71-0.83) of the model discrimination with the sample used to construct the predictive model. It fits under the discrimination category acceptable but almost excellent.^(28)^ The inclination discrimination coefficient was 0.16, close to zero, and Nagelkerke’s R^2^ was 22.71%, indicating that an amount of variation was unexplained and attesting to the multifactorial influences on the outcomes. Figure 1B showed the cut-off point patient of 20.60% chance to be considered high risk.

The predictor model reached high risk of 73.50% accuracy; 65.9% death; 66.50% 30-day readmission, and 71.20% ≥10-day length of stay. The negative predictive value indicated 91.7% of high risk, 100% death, 93.5% 30-day readmission, and 96.3% ≥10-day length of stay.

### Internal validation

Regarding the discrimination obtained with the validation sample, the area under the ROC curve was 0.687 (95%CI: 0.598-0.776), remaining within the considered acceptable discrimination category. The cut-off point would be slightly below that found in the previous model, at 18.5% risk, but still above the cut-off point that supports sensitivity.

## DISCUSSION

Long-term hospitalization, readmission and hospital death are common in the older population, but the clinical intersections of those events are not well known. Understanding the common causes of those conditions may have a significant relevance by the possibility to avoid futures adverse outcomes and consequently higher health costs.

We are not aware of any model that can simultaneously predict these multiple outcomes, considerable gains in effectiveness and efficiency for targeting interventions to patients most likely to benefit. This model allows for early risk stratification and proactive action during hospitalization and discharge planning, providing plan of care which will be delivered by the entire care team, including doctors, nurses, social workers, physiotherapists, dietician, and other professionals.

In the predictive model construction, we obtained an area under the ROC curve of 0.772, meaning acceptable but almost excellent discrimination category. High-risk accuracy measures had a sensitivity of 73.9%; specificity 73.4%; predictive positive value (PPV) 41.5; negative predictive value (NPV) 91.7 and accuracy of 73.5. The high NPV of 91.7, confirm the great probability of the patient who does not configure a risk when the outcome result is negative.

In this study, data collection from medical records was easily performed. And standardized scale scores were applied directly from the patient and family during hospitalization. It has a potential facilitated integration with clinical practice as an aid in decision making support.^(29)^

The association between clinical disease and functional decline had already been described and those circumstance may be present in older patients at hospital settings.^(29)^ This clinical and functional status may act synergically leading to poor health outcomes commonly seeing in hospitalized older adults. The cause consequence relationship between clinical and functional condition may not be so easy to be discriminated, however those situations will lead to a progressive health deterioration if not detected and intervened. In this perspective, a risk stratification tool will help to distinguish those patients which will deserve a customized plan of care aiming to avoid such consequences. Those risk factors associated with adverse prognosis at and after hospital stay will enable the implementation of immediate care interventions during hospitalization, allocation in the geriatric unit and a consistent discharge planning. These findings will allow obtaining the risk factor in a “real time” manner, to initiate some interventions by the interdisciplinary team, during their hospitalization.

The search for an instrument with good accuracy for the screening of older patients still remains, despite the various tools proposed in the literature. Evidence is clear that these risk instruments may reflect a condition of biological inability to react to acute diseases and should be analyzed as a relevant prognostic indicator. After identifying the risk, actions can be implemented, and treatments reviewed.^(6,30)^ In Brazil, not much information is found on how geriatric risk screening followed by comprehensive geriatric assessment (CGA) affects health outcomes of clinical patients hospitalized. In this context, some Brazilian studies cover the risk prediction hospital mortality with adjustment of comorbidity and readmission that contribute to the analysis of hospital care quality for this population.^(12,31,32)^

Due to the complexity of health conditions in this age group, factors such as functional, cognitive, and chewing/swallowing deficiencies, as well as the number of medications impact outcomes. The number of medications showed a direct and independent relationship with the outcomes, considering that there may be a collinearity between the number of medications and diseases. We believe that multimorbidity has influenced the outcomes in this study through the linearity of multimorbidity and number of medications.

Thus, clinical condition (number of medications, chewing/swallowing difficulty, diabetes) associated in a patient with functional and cognition impairment, who underwent extensive surgery, are strongly related to adverse outcomes such as higher length of stay, hospital death, and readmission within 30 days after discharge.

In this context, a CGA may be important to identify subclinical information and establish an appropriate plan of care during and after hospitalization. In addition, in a patient allocation perspective, those older patients at higher risk may benefit from geriatric wards since this vulnerable population may also suffer from other events such as delirium. For those patients, a careful monitoring, surveillance, and action from a multidisciplinary team may have an impact on those vulnerable older adults.

Among tools options, the Barthel Index is as a scale that presents more consistent results and appears to be one of the most useful markers for mortality, readmission, long hospital stays, discharge location, and can predict significant clinical results when evaluating different profiles of older patients.^(14,33,34)^ The present study confirmed it as a useful tool for measuring disability in health and social care settings along the care and treatment.

In Brazil, several studies identified risk factors. However, due to fast aging process, few of those associated these risk factors, such as functional disability or cognition, with the outcomes studied. Barthel is used as a tool to better assess the functionality of older people to quantify functional dependence and understand how hospitalization contributes to functional decline. There are still few scores composed within a multifactorial understanding with a global and practical assistance view.^(14,31,35)^

Many care models focus on the disease and are primarily aimed at reducing it. On the other hand, the possibility to mitigate the clinical adverse outcome after risk stratification is not yet known. Thus, a specific intervention study may be relevant to understand the clinical impact of early risk identification.

This study has some limitations. Since it was conducted in a single private institution, it may raise questions about the general applicability of this predictive model. Private hospitals mainly cover the highest income segment of the population, and it differs from the care provided by Brazilian Public Health System (SUS - *Sistema Único de Saúde*), which has a much higher demand, compromising the access to quality and continuous care.^(36)^

## CONCLUSION

Identifying high-risk patients in real time may act as an early warning system that can lead to timely care interventions and safer transitions.

The present study built and validated a high-risk predictive model with acceptable fit and discrimination for these outcomes: in-hospital death, early readmission up to 30 days after discharge, or long hospital stay (10 days or more).
